# Preparation and Characterization of Resveratrol Loaded Pectin/Alginate Blend Gastro-Resistant Microparticles

**DOI:** 10.3390/molecules23081886

**Published:** 2018-07-28

**Authors:** Oihane Gartziandia, Arrate Lasa, Jose Luis Pedraz, Jonatan Miranda, Maria Puy Portillo, Manoli Igartua, Rosa Maria Hernández

**Affiliations:** 1 NanoBioCel Group, Laboratory of Pharmaceutics, School of Pharmacy, University of the Basque Country (UPV/EHU), Vitoria-Gasteiz 01006, Spain; oihane.gartziandia@ehu.eus (O.G.); joseluis.pedraz@ehu.eus (J.L.P.); manoli.igartua@ehu.eus (M.I.); 2 Biomedical Research Networking Centre in Bioengineering, Biomaterials and Nanomedicine (CIBER-BBN), Vitoria-Gasteiz 01006, Spain; 3 Nutrition and Obesity Group, Department of Nutrition and Food Science, Faculty of Pharmacy and Lucio Lascaray Research Institute, University of País Vasco (UPV/EHU), Vitoria-Gasteiz 01006, Spain; jonatan.miranda@ehu.eus (J.M.); mariapuy.portillo@ehu.es (M.P.P.); 4 CIBEROBN Physiopathology of Obesity and Nutrition, Institute of Health Carlos III (ISCIII), Vitoria-Gasteiz 01006, Spain

**Keywords:** resveratrol, dietary supplement, gastro-resistant, microparticles, obesity, HPLC

## Abstract

Background: The use of resveratrol as a dietary supplement is limited because it is easily oxidized and, after oral ingestion, it is metabolized into enterocytes and hepatocytes. Thus, new formulations are needed in order to improve its oral bioavailability. Objective: The objective of this study was to develop and characterize a gastro-resistant formulation of resveratrol for oral administration as a dietary supplement. Method: Resveratrol was encapsulated in Eudragit-coated pectin-alginate microparticles. Results: The microparticle size was about 1450 µm, with an encapsulation efficiency of 41.72% ± 1.92%. The dissolution assay conducted, as specified in the European Pharmacopoeia for delayed-release dosage forms, revealed that our microparticles were gastro-resistant, because the resveratrol percentage released from microparticles in acid medium was less than 10%. In addition, the high-performance liquid chromatographic (HPLC) method developed for resveratrol content quantification in the microparticles was validated according to International Council for Harmonisation (ICH) Q2 (R1) guidelines. Finally, the biological activity of resveratrol was investigated in 3T3-L1 mature adipocytes, concluding that the encapsulation process does not affect the activity of resveratrol. Conclusion: In summary, the gastro-resistant microparticles developed could represent a suitable method of including resveratrol in dietary supplements and in functional foods used in obesity therapy.

## 1. Introduction

Resveratrol (3,5,4’-trihydroxystilbene), a polyphenolic phytoalexin, is naturally produced in the fruits of several plant species such as grapes, mulberries, and peanuts in response to exogenous stress factors such as injuries, fungal infections, or UV irradiation [1]. It was first found in 1940 in the roots of white hellebore (*Veratrum grandiflorum*). Later, in 1963, it was isolated from the roots of *Polygonum cuspidatum*, a plant used in traditional Chinese medicine. Nowadays, it is mainly extracted from red grapes, whose fresh skin is estimated to contain about 50−100 μg of resveratrol per gram.

**Figure 1:**
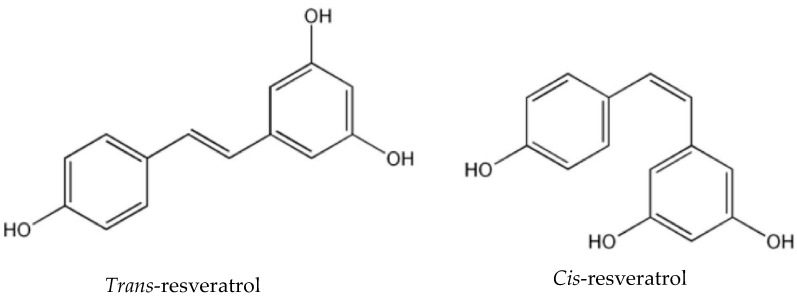
Chemical structure of *trans*- and *cis*-resveratrol.

This polyphenol is a stilbene derivate, which exists in *cis*- and in *trans*- stereoisomeric forms (Figure 1). The most common form in nature, the *trans* form, is relatively stable, but it can undergo isomerisation to the *cis*- form when exposed to ultraviolet irradiation [2]. This isomerisation is not desirable, because the *trans-* form is the steric form, which is responsible for the beneficial effects of this compound. Therefore, it is important to maintain its stability in order to retain its biological and pharmacological activities [1]. 

The pharmacological activity of resveratrol takes the form of an antioxidant and anti-inflammatory effect on a wide range of disorders associated with inflammation, such as diabetes mellitus, obesity, cardiovascular disease, neurodegenerative diseases, or cancer [2]. Currently, resveratrol is mainly used as an antioxidant dietary supplement to protect against cardiovascular problems and some alterations associated with aging, and as a supplement in the treatment of cancer, obesity, diabetes, or hypercholesterolemia [3]. The doses of resveratrol used in these supplements range from 50 to 500 mg per capsule.

Nevertheless, the use of resveratrol is limited by its being easily oxidable and extremely photosensitive. In addition, its low solubility in water and its rapid metabolism in enterocytes and hepatocytes (where sulphate and glucuronic conjugates are produced), as well as its rapid elimination, make the oral resveratrol bioavailability very low [4,5]. As a result, several strategies are being carried out to increase its bioavailability, such as gastro-resistant microparticles that delay release until it reaches the distal portions of the intestinal tract [6].

Microencapsulation consists of coating drugs with different materials to obtain particles of micrometric size. It is a commonly used technique in pharmaceutical, cosmetic, and food industries, because it increases the stability of the active component and the release of the drug is controlled [1].

In this context, pectin (a natural polysaccharide) may be considered as a suitable substance for the development of gastro-resistant microparticles, as it is specifically degraded by pectinolytic enzymes produced by colonic microbiota, while resisting enzymes present in the stomach and intestine. This natural polysaccharide is composed of partially methoxylated poly (1-4-*α*-D-galacturonic acids) with some 1–2 linked L-rhamnose groups [7]. It is present in the cell wall of most edible plants, and so it is approved for its use as an excipient in oral formulations [8]. Moreover, pectins with low levels of methoxylation can be cross-linked with calcium ions (Ca^2+^, divalent cations) to produce Ca-pectinate networks, therefore, these have been used as vehicles for drug delivery [9].

In addition to pectin, alginate has also been used for the development of microparticles because of its bio/mucoadhesive properties. Thus, the preparation of ca-pectinate/alginate microparticles is an interesting approach, because pectin can protect from enzymatic digestion and alginate can prolong the residence time of particles in the administration site, and so achieve controlled drug release [10]. However, the use of pectin and alginate as the sole components of microparticles is not enough to completely avoid resveratrol release until it reaches the distal intestinal tract. For this reason, in order to obtain gastro-resistant microparticles, they were coated with Eudragit^®^ FS-30D, a methacrylic acid copolymer that dissolves at pH 7. This polymer avoids the dissolution of the microparticles in the first portions of the gastrointestinal tract, and the consequent release of the drug in the portions of the intestine with a pH ≥ 7 [11].

With these considerations in mind, the objective of this study was to develop and characterize resveratrol loaded pectin/alginate blend gastro-resistant microparticles to be orally administered as a dietary supplement. 

## 2. Results

### 2.1. Validation of the Quantification Technique of Resveratrol by High-Performance Liquid Chromatographic (HPLC)

Following ICH Q2(R1) guidelines, we have demonstrated that our method meets the linearity criteria described in Table 1. The results show an excellent correlation between the areas of the chromatographic peak and the concentration of the analyte in the concentration range of 5–60 µg/mL.

We also demonstrated that our method was selective, precise, accurate, and specific because all parameters met the established acceptance criteria (Table 1).

In addition, we proved that although the resveratrol standard solution was stable for 96 h at room temperature, the sample test solution was only stable for 24 h, taking into account the established acceptance criteria (Table 1).

### 2.2. Particle Size and Morphology

After measuring the diameter of randomly selected fifty dry microparticles, as expected, the mean particle size was 1443.45 µm ± 126.11 µm, as we expected, as a result of using the ionotropic gelation method with a 25G needle to prepare the microparticles [9].

**Figure 2:**
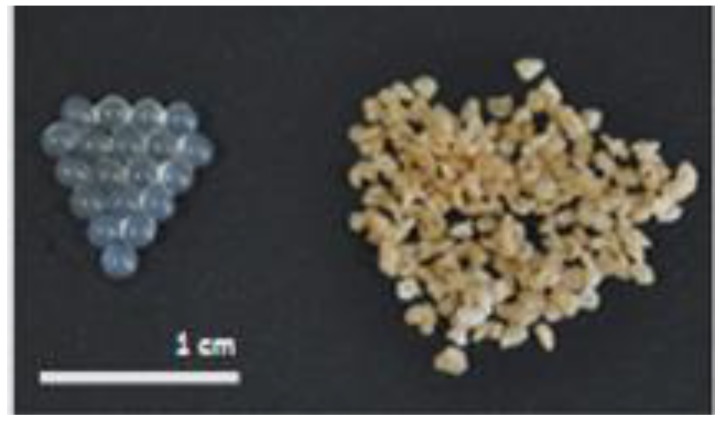
Comparison of morphology and particle size between newly prepared (left) and dried (right) microparticles.

In terms of morphology, the newly prepared microparticles showed a spherical and uniform shape. However, with dehydration the morphology became more irregular, and particle size was reduced by about 500 µm, from 2000 to 1500 µm (Figure 2).

### 2.3. Encapsulation Efficiency (EE)

EE (%) determined by HPLC was 41.72% ± 1.92%. The low EE was most probably linked to the lipophility of resveratrol, which is why during gelation resveratrol could undergo diffusion from pectin-alginate mixture to gelation solution (CaCl_2_). Similar EE results were obtained by other authors using the same method for encapsulation of Cwp84 antigen [12].

### 2.4. Drug Release Study: Resveratrol Release from Microparticles

**Figure 3:**
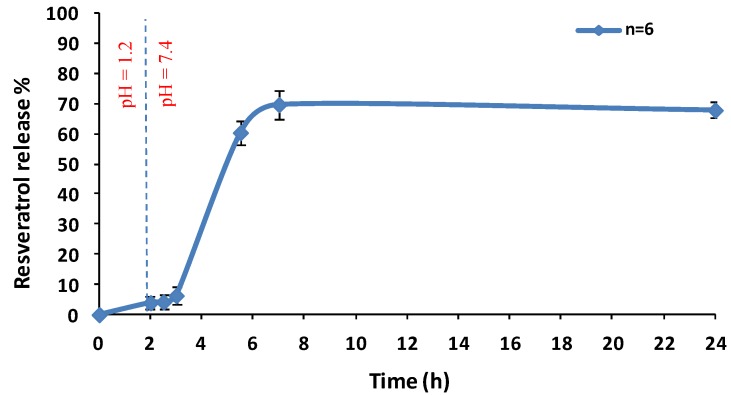
Percentage of resveratrol release from microparticles. The microparticles were dispersed in 750 mL of an acid medium (pH 1.2) for 2 h, followed by a basic medium (pH 7.4). Values represent the means (*n* = 6 for each test) ± the standard error.

As we can see in the Figure 3, in the first 2 h of the study, when particles were in contact with acidic medium, the percentage of resveratrol released from microparticles was less than 10%, meeting the criteria established in the European Pharmacopoeia monograph for gastro-resistant formulations [13]. After 2 h, when the medium pH was changed from acidic to basic, the microspheres showed a faster drug release rate, until reaching 70% of resveratrol total content in 24 h.

### 2.5. In Vitro Bioactivity Study: Measurement of Triacylglycerol Content in Adipocytes

**Figure 4:**
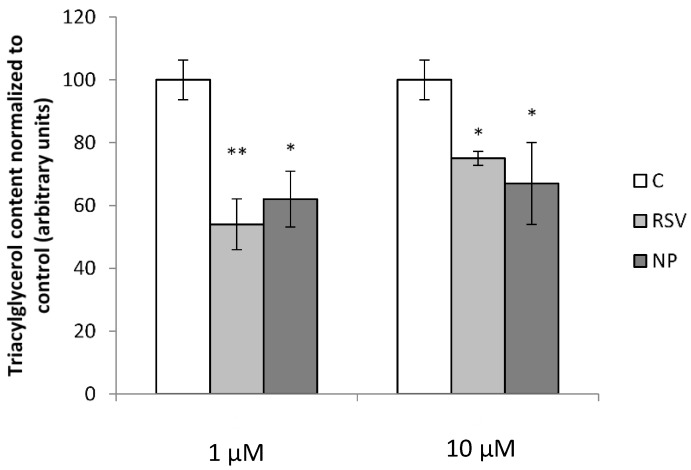
Triacylglycerol content in 3T3-L1 mature adipocytes after treatment with 1 and 10 µM free resveratrol (RSV) and RSV released from microparticles (NP). Values are means ± SEM. Comparison between each treatment with the control was analyzed by Student’s *t*-test. The asterisks represent differences versus the control (* *p* < 0.05; ** *p* < 0.01).

After incubation of adipocytes with resveratrol for 24 h, a reduction in triacylglycerol content was observed for both concentrations used (1 and 10 µM). However, a dose-dependent effect was not observed. When comparing the effect of resveratrol released from the microparticles with that of free resveratrol, no differences were found between both compounds (Figure 4).

## 3. Discussion

In the present study resveratrol loaded pectin/alginate blend gastro-resistant microparticles were prepared as a new formulation to improve the oral bioavailability of resveratrol. Pectin resists the enzymes present in the stomach and intestine, so it could protect resveratrol from metabolism until it reaches the more distal parts of the intestine. Furthermore, alginate has properties which can prolong the residence time of the microparticles in the administration site, and achieve a controlled release, thanks to its bio/mucoadhesive properties [10]. The combination of these compounds is a good strategy for the development of microparticles because they can be cross-linked with calcium ions (Ca^2+^, divalent cations) obtaining Ca-pectinate-Ca-alginate networks. Therefore, the ionotropic gelation method has been used for the preparation of pectin/alginate blend microparticles [9]. The resulting microparticles were subsequently coated with the enteric polymer Eudragit® FS-30D to avoid completely the release of resveratrol until it reaches the distal intestinal tract, since this methacrylic acid copolymer dissolves to pH 7 [11].

In order to demonstrate that we succeeded in producing gastro-resistant microparticles, we carried out the dissolution assay described in the European Pharmacopoeia for delayed-release dosage forms [14]. As the release study revealed, when particles were in contact with acid medium, the percentage of resveratrol released from microparticles was less than 10%, meeting the criteria established in the European Pharmacopoeia monograph for gastro-resistant formulations. While when the pH of the medium was changed from acid to basic (pH 7.4), a faster dissolution rate which reached 70% of the total content in 24 h was observed. These results demonstrated that Eudragit FS-30D enteric coating protected resveratrol loaded microparticles from acid pH, and therefore, that resveratrol will be released into the distal portions of the intestinal tract after oral ingestion of miroparticles [15]. 

The advantages of microencapsulation to protect resveratrol and to increase its bioavailability have been described in this manuscript, but when this strategy is used, it is necessary to check whether it affects the biological activities of this compound. For his purpose, in the present study the effects of encapsulated resveratrol and free resveratrol (used as a control) on triacylglycerol content in 3T3-L1 mature adipocytes was analyzed because, according to the results obtained in in vitro and in vivo studies [16,17,18], resveratrol has been proposed as a potential anti-obesity compound. It seems to mimic the effects of energy restriction, thus leading to reduced body fat and improved insulin sensitivity [19]. Resveratrol supplements usually provide mg of this compound [20,21,22]. After absorption, intestinal and hepatic metabolism, the amounts of resveratrol found in plasma and tissues are in the range of 1–2 μM [23,24]. Taking this is mind, the amounts of resveratrol used in the present study for adipocyte culture were 1 and 10 μM.

Both forms of resveratrol, free and encapsulated, reduced triacylglycerol content at the two doses used, and no differences were observed between them, meaning that in fact resveratrol was not negatively affected by microencapsulation. In both cases the effect of 1 μM was greater than that of 10 μM comparing to control. Although this seems surprising, the relationship between the dose and the effectiveness is quite complex in polyphenol studies. In the case of resveratrol, this situation (greater effects of lower doses than those of higher doses) has been already found in in vivo studies by other authors. Thus, Cho et al., observed greater anti-obesity effect of resveratrol when mice were treated with this phenolic compound at a dose of 0.005% in the diet than when they were treated at a dose of 0.02% [25].

Taken as a whole, the present results show that pectin/alginate gastro-resistant microparticles protect resveratrol from acid pH, allowing the phenolic compound to be released into the distal portions of the intestinal tract after oral ingestion of miroparticles. In addition, resveratrol remains active after the encapsulation process. Consequently, this type of microencapsulation could be a useful strategy in the development of dietary supplements or functional foods that may be beneficial for the prevention or treatment of obesity. 

## 4. Materials and Methods

### 4.1. Materials

Pectin (from apple) (CAS Number: 9000-69-5), alginate (from Brown algae) (CAS Number: 9005-38-3), calcium chloride (CAS Number: 10043-52-4), and pectinase (from Aspergillus aculeatus) (MDL number: MFCD00131809) were purchased from Sigma-Aldrich Química S.A (Madrid, Spain). *Trans*-resveratrol (Resveratrol 95% (HPLC) from Polygonum cuspidatum) was supplied by Monteloeder (Alicante, Spain). Eudragit FS-30D was obtained from Evonik (Essen, Alemania). Triethyl citrate (TEC) (CAS Number: 77-93-0) was donated by Morflex. Methanol HPLC grade (CAS Number: 67-56-1), Formic acid (98–100%, reagent grade) (CAS Number: 64-18-6), HCl 35% *w/w* (CAS Number: 7647-01-0), NaOH (CAS Number: 1310-73-2) and tri-Sodium phosphate monohydrate were purchased from Scharlau (Barcelona, Spain). Dulbecco´s modified Eagle´s medium (CAS Number: 103130-21) was supplied by GIBCO (BRL Life Technologies, Grand Island, NY, USA). Triacylglcyerols (TG) were determined by Infinity Triglycerides reagent (Thermo Electron Corporation, Rockford, IL, USA) and protein concentrations of cell extracts were measured with BCA reagent (Thermo Scientific, Rockford, IL, USA). 

### 4.2. Preparation of Microparticles

Microparticles were prepared using the ionotropic gelation method [9]. Firstly, pectin (2%, (*w/v*); 2 g in 100 mL) and alginate (1%, (*w/v*); 1 g in 100 mL) were dissolved in deionized water and resveratrol (10%, *w/w*; 0.3 g) was dispersed in it. This mixture was then added dropwise through a needle of 25G on a gently agitated CaCl_2_ dissolution (5%, (*w/v*); 10 g in 200 mL), using a peristaltic pump (ecoline, ISMATEC, 0.4 mL/min). Subsequently, microparticles were obtained by the gelation of the pectin and the alginate and held in magnetic stirring for 3 h. The microparticles were then separated; washed with distilled water; and dried on a fluidized bed, Mini Glatt 4^®^ (for 10 min at 70 °C).

The dried microparticles were coated with a Eudragit^®^ FS-30D (20%, (*w/w*)) and TEC (1.5%, (*v/w*)) solution, on the fluidized bed, Glatt^®^ (70 °C, 0.6–0.7 bar). The coating solution was atomized into microparticles using a peristaltic pump (0.3 mL/min) until the desired coating thicknesses were achieved, with an increase in weight of 20%.

Empty microparticles were prepared by the same procedure, but without adding resveratrol to the pectin and alginate mixture.

### 4.3. Development and Validation of an HPLC Method for the Quantification of Resveratrol

Quantitative high-performance liquid chromatographic (HPLC) analysis was performed on a Waters HPLC system (Waters Corporation, Milford, MA, USA) equipped with a binary HPLC pump (Waters 1525), a dual λ Absorbance UV-visible detector (Waters 2487), a column oven (Waters Column Heater Module), and an auto sampler (Waters 717 plus Autosampler) controlled by software (Empower 3 Software), which was used for data analysis and processing.

An HPLC method was developed for the quantification of resveratrol in the microparticles. It was carried out on a XBridge column (4.6 × 75 mm, C18 2.5 μm) with column oven temperature of 35 °C using a gradient elution system consisting of MeOH and Formic acid 0.1%. The proportion of Formic acid/MeOH in the gradient was 50:50, 50:50, 10:90, 10:90, 50:50, and 50:50, at 0, 0.5, 2, 4.5, 4.51, and 8.5 minutes, respectively. The retention time of *trans*- and *cis*-resveratrol were 2.42 min and 3.84 min, respectively, with a flow rate of 0.7 mL/min. The analysis was carried out at 305 nm and 285 nm wavelength for *trans*- and *cis*-resveratrol, respectively, with a run time of 8.5 min.

The method was validated according to the ICH Q2(R1) guideline [13] in terms of linearity in the concentration range of 5–60 µg/mL, selectivity, precision, accuracy, specificity and stability. With the results obtained in each of the trials, it was seen whether the different parameters met the established acceptance criteria, described in Table 1.

### 4.4. Characterization of Microparticles

#### 4.4.1. Particle Size

The size of the dry microparticles was measured by an optical microscope (Nikon ECLIPSE TE2000-S), set at 4X objective. Fifty microparticles were randomly selected, and the diameter of each one was measured with the help of the scale of the Eclipse Net software, after capturing the images through a digital camera (Nikon Digital Sight DS-U1) connected with the microscope. The average size of the microparticles was expressed as the mean diameter (µm) ± standard deviation (SD).

#### 4.4.2. Encapsulation Efficiency (EE%)

Resveratrol EE was determined by a direct method. Briefly, 10 mg of microparticles was added to a topaz flask of 10 mL with a mix of MeOH–H_2_O–Formic acid (45:55:0.1) and pectinase (1%, *w/v*). The mixture was left in magnetic stirring until the dissolution of the particles, in order to completely extract the resveratrol from microparticles. The concentration of resveratrol in the flask was determined by HPLC. The experiment was performed in triplicate. EE was expressed as the actual resveratrol mass percentage, compared with the total mass of resveratrol added initially.

#### 4.4.3. Dissolution Assay

The assay was conducted as described in the European Pharmacopoeia for delayed-release dosage forms under Apparatus 1 (Sotax dissolution tester), using the specified media at 37 °C and 50 rpm [14].

Firstly, the test was performed in an acid medium (HCl, 0.1 N). For this, seven vessels of the apparatus were filled with 750 mL of 0.1 N hydrochloric acid, and microparticles were placed in the baskets (empty microparticles in the first vessel, and resveratrol loaded in the rest). After 2 h of operation, an aliquot of each vessel was withdrawn and replaced with the same volume of medium. 

Immediately, 250 mL of tri-Sodium phosphate monohydrate was added to the vessels and the pH was adjusted to 7.4 with NaOH 2 N, to continue with the test in a basic medium. At different times (1 h, 2.5 h, 5 h, and 24 h) aliquots were withdrawn and replaced with the same volume of medium.

All aliquots were analyzed by HPLC to obtain the concentration of resveratrol, and results were expressed as cumulative percentage of resveratrol released from the microparticles at a given time, against the initial resveratrol loading in the microparticle sample.

### 4.5. In Vitro Bioactivity Assay

#### 4.5.1. Experimental Design

The 3T3-L1 pre-adipocytes, supplied by American Type Culture Collection (Manassas, VA, USA), were cultured in DMEM containing 10% foetal calf serum (FCS). Two days after confluence (day 0), the cells were stimulated to differentiation with DMEM containing 10% *v/v* FCS, 10 µg/mL insulin, 0.5 mM isobutylmethylxanthine (IBMX), and 1 μM dexamethasone for two days. On day 4, the differentiation medium was replaced by FBS/DMEM medium (10%) containing 0.2 µg/mL insulin. This medium was changed every two days until cells were harvested. All media contained 1% *v/v* Penicillin/Streptomycin (10,000 U/mL), and the media for differentiation and maturation contained 1% (*v/v*) of Biotin and Panthothenic Acid. Cells were maintained at 37 °C in a humidified 5% CO_2_ atmosphere. 

#### 4.5.2. Cell Treatment

For the treatment of mature adipocytes, cells grown in six-well plates were incubated with free resveratrol and resveratrol released from microparticles, at 1 and 10 μM (diluted in 95% ethanol, final ethanol concentration in the medium 0.1%) on day 12 after differentiation because on that day, >90% of cells had matured, with visible lipid droplets. After 24 h, supernatant was removed and cells were used for triglyceride (TG) determination. This experiment was repeated three times.

#### 4.5.3. Measurement of Triacylglycerol Content in Adipocytes

Mature adipocytes were washed extensively with phosphate-buffered saline (PBS) and incubated three times with 800 µL of hexane/isopropanol (2:1). The total volume was then evaporated by nitrogen gas and the pellet was resuspended in 200 µL Tritón X-100 in 1% distilled water. Afterwards, TG were disrupted by sonication and the content was measured by a commercial kit. For protein determinations, cells were lysed in 0.3 N NaOH, 0.1% SDS. Protein measurements were performed using the BCA reagent. TG content results were obtained as mmol glycerol/mg protein and were converted to arbitrary units.

#### 4.5.4. Statistical Analysis

Results are presented as mean + standard error of the mean. Statistical analysis was performed using SPSS 19.0 (SPSS Inc., Chicago, IL, USA). Statistical analysis was determined by unpaired Student’s unpaired *t*-test (two-tailed). Statistical significance was set-up at the *p* < 0.05 level.

## 5. Conclusions

Taking into account the results obtained, it may be concluded that the developed gastro-resistant microparticles can represent an appropriate strategy for the inclusion of resveratrol in dietary supplements and functional foods with potential beneficial effects in the prevention or treatment of obesity. 

## Figures and Tables

**Table 1:** Acceptance criteria.

Parameter		Especification	
Selectivity		Identification	-
		Resolution	Rs > 1.5
		Absence of interference	no interferences
Linearity		Correlation coefficient	r ≥ 0.999
		C.V. response factors	C.V. ≤ 2%
		Relative error percentage	≤2%
		Slope linearity test	t_exp_ > t_table_
		Slope confidence intervals	No include 0
		Test of proportionality	t_exp_ < t_table_
		Intercept confidence intervals	Include 0
Repeatability of Instrumental System		C.V.	C.V. < 1.37%
Repeatability of the Method		C.V.	C.V. < 1.94%
Intermediate Precision		C.V. individuals	C.V. < 1.94%
		C.V. intermediate	C.V. < 3.88%
Recovery percentage		Recovery	98.0–102.0%
		Relative error percentage	≤2%
		Test for equality of variances	G_exp_ < G_table_
Robustness	Influence of analyst	C.V.	C.V. < 3.88%
	Influence of analysis temperature	C.V.	C.V. < 1.94%
Stability	Standard	Concentration in relation to time 0	98.0–102.0%
	Sample		

C.V.: Coefficient of variation; G_exp_: Experimental G value; G_table_: G value in tables.
